# CEUS for the diagnosis of acute graft pyelonephritis in renal transplant recipients

**DOI:** 10.1007/s40477-026-01228-7

**Published:** 2026-09-26

**Authors:** Andrea Boccatonda, Alice Brighenti, Valeria Tiraferri, Marco Musmeci, Daniela Agostinelli, Nicola Venturoli, Livia Masi, Attilio Barretta, Elisa Gessaroli, Giorgia Comai, Carla Serra

**Affiliations:** 1 Diagnostic and Therapeutic Interventional Ultrasound Unit, IRCCS Azienda Ospedaliero-Universitaria di Bologna, Policlinico Sant’Orsola-Malpighi, via Massarenti n 9, 40138 Bologna, Italy; 2 Nephrology, Dialysis and Kidney Transplant Unit, IRCCS Azienda Ospedaliero-Universitaria di Bologna, Bologna, Italy; 3 Division of Urology, IRCCS Azienda Ospedaliero-Universitaria di Bologna, Bologna, Italy

**Keywords:** Ultrasound, CEUS, Pyelonephritis, Urinary infection, Kidney transplant

## Abstract

**Purpose:**

Acute graft pyelonephritis (AGPN) is a clinically relevant complication in renal transplant recipients, in whom diagnosis may be challenging because of nonspecific clinical and conventional ultrasound findings. Contrast-enhanced ultrasound (CEUS) enables real-time assessment of renal microvascular perfusion without nephrotoxic contrast agents. This study aimed to evaluate the diagnostic performance of CEUS for detecting AGPN in renal transplant recipients.

**Methods:**

This retrospective single-center study included 66 consecutive renal transplant recipients who underwent CEUS for clinically suspected AGPN. The final clinical diagnosis, based on the integration of clinical, laboratory, microbiological, and available imaging findings, was used as the reference standard. Conventional ultrasound findings and CEUS parenchymal perfusion abnormalities were assessed. Diagnostic performance was evaluated in terms of sensitivity, specificity, positive predictive value (PPV), negative predictive value (NPV), and likelihood ratios.

**Results:**

AGPN was confirmed in 21/66 patients (31.8%). Fever was more frequent in patients with AGPN than in those without AGPN (100% vs. 73.3%, p=0.014). On conventional ultrasound, globular graft appearance (14.3% vs. 0%, p=0.029), cortical hyperechogenicity (23.8% vs. 0%, p=0.002), cortical hypoechogenicity (28.6% vs. 4.4%, p=0.010), perirenal fluid collection (38.1% vs. 11.1%, p=0.018), and ureteral wall thickening (42.9% vs. 8.9%, p=0.002) were significantly associated with AGPN. CEUS correctly identified 19/21 AGPN cases, with two false-negative and no false-positive examinations. Sensitivity was 90.5% (95% CI 69.6–98.8%), specificity 100% (95% CI 92.1–100%), PPV 100% (95% CI 82.4–100%), and NPV 95.7% (95% CI 85.5–99.5%).

**Conclusion:**

CEUS showed high diagnostic accuracy for AGPN in renal transplant recipients. By providing real-time assessment of graft parenchymal perfusion without nephrotoxicity or ionizing radiation, CEUS may represent a valuable complement to conventional ultrasound and potentially reduce the need for contrast-enhanced CT in selected patients. Larger prospective multicenter studies with standardized independent reference standards are warranted.

## Introduction

Renal transplant recipients (RTRs) are characterized by an increased risk of infectious complications due to chronic immunosuppression and post-surgical anatomical changes. Urinary tract infections (UTIs) represent the most frequent infectious complication, with reported incidence ranging from approximately 28% to 38% over time [1–4]. Their occurrence is particularly high during the early post-transplant period, especially within the first 3–6 months, when multiple risk factors coexist, including ureteral stenting, indwelling catheters, and surgical manipulation [5–7]. In this population, UTIs may extend to involve the renal graft, resulting in acute graft pyelonephritis, a clinically significant condition associated with graft dysfunction, increased morbidity, and potential impact on long-term graft survival [5–7]. The diagnosis of acute graft pyelonephritis is often challenging, as clinical presentation may be atypical and non-specific due to immunosuppression, and differentiation between graft involvement and lower UTIs is not always straightforward. From a microbiological standpoint, Gram-negative bacilli are responsible for the majority of infections in RTRs, frequently involving multidrug-resistant organisms that complicate empirical antibiotic therapy and worsen clinical outcomes [6, 8]. Imaging therefore plays a crucial role in the diagnostic workup of suspected acute graft pyelonephritis. Contrast-enhanced computed tomography (CECT) is widely used but is limited in RTRs by the potential nephrotoxicity of iodinated contrast agents, which may compromise graft function [3, 9]. Contrast-enhanced ultrasound (CEUS), based on intravascular microbubble contrast agents, may represent a safe and non-nephrotoxic imaging modality that allows real-time evaluation of renal perfusion [3, 9]. Despite its theoretical advantages, evidence regarding its diagnostic performance in RTRs with suspected acute graft pyelonephritis remains limited.

## Study aims

The main aim of this study was to evaluate the diagnostic accuracy of CEUS in detecting acute graft pyelonephritis in RTRs, using the final clinical diagnosis as the reference standard. Secondary objectives included the assessment of sensitivity, specificity, positive and negative predictive values, and likelihood ratios of CEUS in this clinical setting. In addition, we aimed to describe conventional ultrasound and CEUS imaging features associated with acute graft pyelonephritis and to characterize the microbiological profile of confirmed cases.

## Materials and methods

### Study design and population

This was a retrospective, observational, single-center study conducted at IRCCS Sant’Orsola-Malpighi Hospital (Bologna, Italy). Consecutive RTRs who underwent CEUS for clinically suspected acute graft pyelonephritis between January and December 2024 were included. Inclusion criteria were adult patients (≥ 18 years) with clinical suspicion of acute pyelonephritis involving the renal graft. Exclusion criteria included pregnancy, pediatric age, and known contraindications to ultrasound contrast agents. In particular, patients with known hypersensitivity to sulfur hexafluoride or any component of the contrast agent, as well as those with severe cardiopulmonary conditions (including unstable ischemic heart disease, recent acute coronary syndrome, or severe pulmonary hypertension), were excluded in accordance with current safety recommendations for contrast agent administration. The study was conducted according to the local institutional protocol and internal clinical practice regulations. The study was conducted in accordance with the Declaration of Helsinki and approved by the local Institutional Review Board (CE AVEC: 832/2022/Sper/AOUBo).

### Pyelonephritis reference standard

The reference standard was the final clinical diagnosis established at discharge, based on the integration of clinical presentation, laboratory findings, microbiological data, and imaging findings when available. CECT findings were recorded when performed but were not mandatory for diagnosis. The physician establishing the final clinical diagnosis was different from the physician who performed and interpreted the ultrasound and CEUS examinations. However, owing to the retrospective nature of the study and routine clinical workflow, clinicians establishing the final diagnosis were not systematically blinded to CEUS findings.

### Clinical and laboratory data collection

Clinical data were retrospectively collected from electronic medical records. Demographic variables comprised age and sex. Clinical characteristics included comorbidities, presenting symptoms (fever, flank pain, urinary symptoms), and relevant medical history. Laboratory parameters consisted of inflammatory markers such as C-reactive protein (CRP) and procalcitonin (PCT) when available, white blood cell (WBC) count and renal function parameters (serum creatinine). Microbiological data were recorded, including isolated pathogens from urine cultures and blood cultures, and their distribution within the study population (Table 1).

**Table 1:** Baseline demographic and clinical characteristics of the study population

	n (%)
Age	56.2 ± 12.9
Sex (Male)	41 (62.1%)
Renal Stenting	26 (39.4%)
Nephrostomy	1 (1.5%)
Type 2 Diabetes	20 (30.3%)
Heart failure	11 (16.6%)
Hypothyroidism	4 (6.0%)
Immunodepression	65 (98.5%)
Fever (> 38°C)	55 (83.3%)
Flank pain	12 (18.2%)
Dysuria	23 (34.8%)

Data are presented as mean ± standard deviation for continuous variables and as number (percentage) for categorical variables. Variables include age, sex, comorbidities, transplant-related factors, and presenting clinical symptoms at admission

### Ultrasound and CEUS examination

**Fig. 1:**
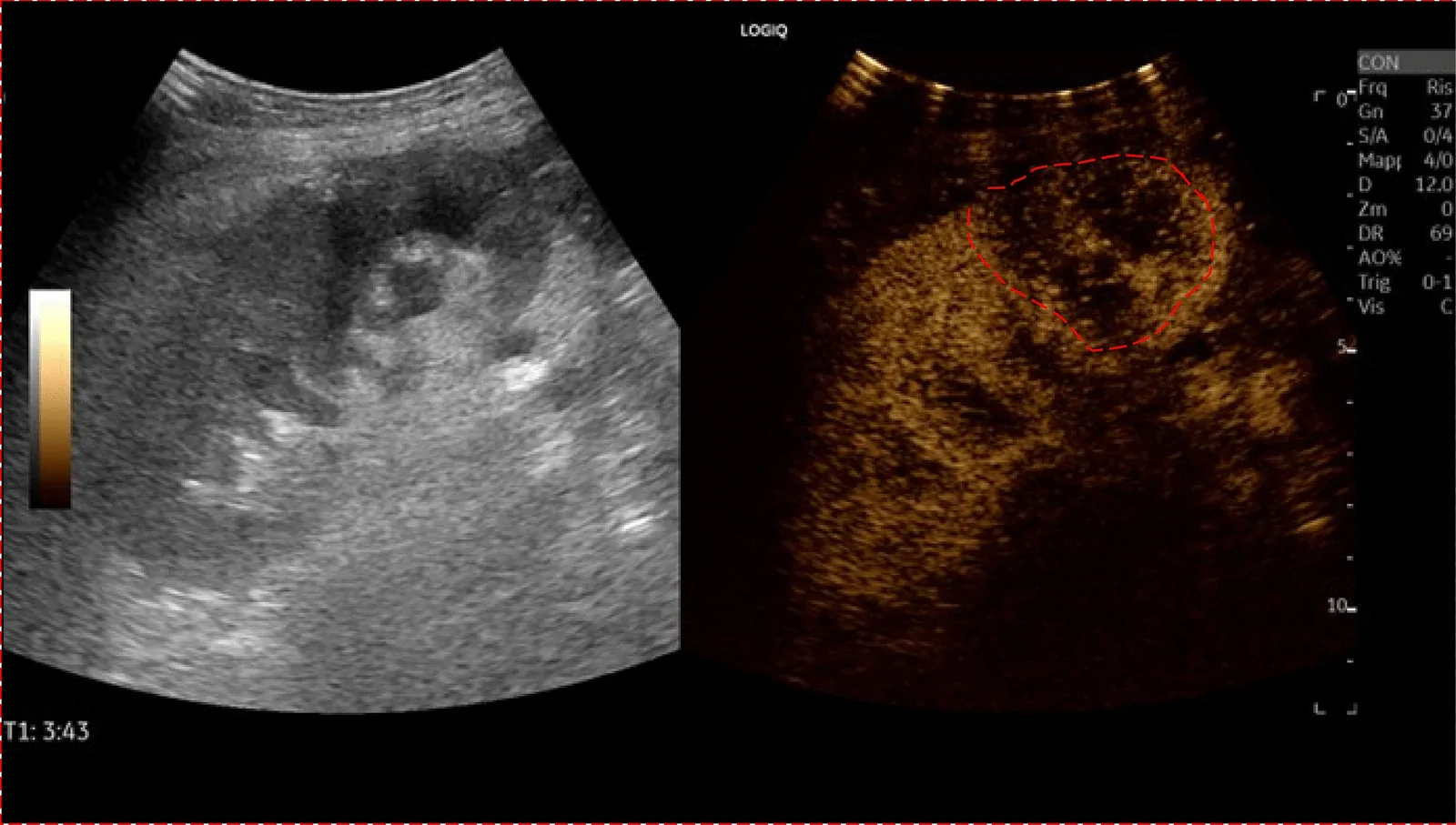
B-mode ultrasound (left) and contrast-enhanced ultrasound (CEUS, right) of a renal graft in a patient with acute graft pyelonephritis. While B-mode imaging shows non-specific parenchymal changes, CEUS clearly demonstrates a focal cortical perfusion defect (outlined by the red dashed line) in late phase, consistent with inflammatory parenchymal involvement

All ultrasound examinations were performed using a high-end ultrasound system equipped with contrast-specific imaging software. A convex transducer with a frequency range of approximately 1.0–5.0 MHz was used. The renal graft was systematically evaluated using both axial and longitudinal scans. Baseline B-mode ultrasound was performed to assess renal morphology and to identify indirect signs suggestive of infection, including alterations in cortical echogenicity, changes in renal size, hydronephrosis, perirenal fluid collections, and ureteral abnormalities. Globular appearance was qualitatively defined as a more rounded and swollen configuration of the renal graft, suggestive of parenchymal edema, compared with its usual reniform morphology; no predefined dimensional cut-off was applied. A duplex Doppler evaluation was also performed in all cases to assess vascularization. CEUS was subsequently performed in all patients with clinical suspicion of acute pyelonephritis. A second-generation ultrasound contrast agent (SonoVue®, Bracco Imaging, Milan, Italy), consisting of sulfur hexafluoride microbubbles stabilized by a phospholipid shell, was administered intravenously via a peripheral venous access (≥ 20-gauge), with the shortest possible line length to minimize microbubble disruption. A bolus of 2.4 mL was injected at a rate of approximately 1–2 mL/s, followed by a 5–10 mL saline flush at approximately 2 mL/s. Contrast-specific imaging was performed using continuous low- Mechanical Index (MI) acquisition, with the MI maintained below 0.10, to minimize microbubble disruption and enable real-time assessment of renal microvascular perfusion. The examination protocol was consistent with current international recommendations for CEUS imaging. CEUS findings suggestive of acute pyelonephritis included the presence of focal or multifocal perfusion defects within the renal parenchyma, predominantly involving the cortex and, in some cases, extending into the medullary region. These lesions typically appeared as wedge-shaped or rounded areas of reduced or absent enhancement compared with the surrounding parenchyma. Lesions were more clearly detectable during the late parenchymal phase, whereas earlier phases often showed more heterogeneous or less well-defined enhancement patterns (Fig. 1). In some cases, lesions demonstrate dynamic enhancement behavior, with initial hypoenhancement followed by transient isoechogenicity and subsequent return to hypoenhancement. When identifiable, lesions were categorized as unifocal or multifocal according to their distribution within the graft. All CEUS examinations were performed and interpreted by a single experienced operator in routine clinical practice.

## Statistical analysis

Continuous variables were expressed as mean ± standard deviation (SD), while categorical variables were reported as frequencies and percentages. Diagnostic performance of CEUS was assessed using 2×2 contingency tables, calculating sensitivity, specificity, positive predictive value (PPV), negative predictive value (NPV), and likelihood ratios (LR+ and LR −), with corresponding 95% confidence intervals. Receiver operating characteristic (ROC) curve analysis was performed, and the area under the curve (AUC) was calculated to evaluate overall diagnostic performance. A p-value < 0.05 was considered statistically significant. Statistical analysis was performed using SPSS software.

## Results

A total of 66 renal transplant recipients were included in the study. Acute graft pyelonephritis (AGPN) was confirmed in 21 patients (31.8%), whereas 45 patients did not receive a final diagnosis of AGPN. The two groups were broadly comparable in terms of baseline demographic and clinical characteristics (Table 2). Mean age was 54.0 ± 12.8 years in the AGPN group and 57.5 ± 13.0 years in the non-AGPN group (p = 0.309). Male sex was more frequent among patients with AGPN (81.0% vs. 53.3%), although the difference did not reach statistical significance (p = 0.055). No statistically significant differences were observed in comorbidities, including type 2 diabetes, heart failure, hypothyroidism, and hypertension, or in transplant-related factors such as the presence of ureteral stents or nephrostomy.

**Table 2:** Comparison of baseline demographic and clinical characteristics between patients with acute graft pyelonephritis (AGPN) and those without AGPN

Variable	AGPN (N=21)	No AGPN (N=45)	P-Value
Age, Years (Mean ± Sd)	54.0 ± 12.8	57.5 ± 13.0	0.309
Male sex, N (%)	17 (81.0%)	24 (53.3%)	0.055
Renal stent, N (%)	11 (52.4%)	15 (33.3%)	0.180
Nephrostomy, N (%)	0 (0.0%)	1 (2.2%)	1.000
Type 2 diabetes, n (%)	7 (33.3%)	13 (28.9%)	0.777
Heart Failure, N (%)	5 (23.8%)	6 (13.3%)	0.307
Hypothyroidism, N (%)	2 (9.5%)	2 (4.4%)	0.587
Immunosuppression, N (%)	21 (100.0%)	44 (97.8%)	1.000
Fever >38 °C, N (%)	21 (100.0%)	33 (73.3%)	0.014
Flank Pain, N (%)	6 (28.6%)	6 (13.3%)	0.175
Urinary Symptoms, N (%)	10 (47.6%)	13 (28.9%)	0.170
Hypertension, N (%)	10 (47.6%)	23 (51.1%)	1.000
Days after symptom onset (Mean ± SD)	6.7 ± 4.1	6.0 ± 5.2	0.557
Recurrent Pyelonephritis, N (%)	5 (23.8%)	4 (8.9%)	0.130

Continuous variables are presented as mean ± standard deviation, and categorical variables as number (percentage)

Fever was significantly more frequent in patients with AGPN than in those without AGPN (100.0% vs. 73.3%, p = 0.014). Flank pain (28.6% vs. 13.3%, p = 0.175) and urinary symptoms (47.6% vs. 28.9%, p = 0.170) were numerically more frequent in the AGPN group but did not differ significantly between groups. The time interval between symptom onset and clinical evaluation was also similar (6.7 ± 4.1 vs. 6.0 ± 5.2 days, p = 0.557), as was the frequency of recurrent pyelonephritis (23.8% vs. 8.9%, p = 0.130).

Laboratory findings are reported in Table 3. Patients with AGPN showed significantly higher C-reactive protein levels than patients without AGPN (11.2 ± 10.3 vs. 5.6 ± 6.3 mg/L, p = 0.016). Procalcitonin levels were higher in the AGPN group, although the difference did not reach statistical significance (10.9 ± 23.1 vs. 2.8 ± 7.3 ng/mL, p = 0.094). No significant differences were observed in white blood cell count or serum creatinine levels between the two groups.

**Table 3:** Comparison of laboratory findings between patients with acute graft pyelonephritis (AGPN) and those without AGPN

	AGPN (n=21)	No AGPN (n=45)	P-Value
CRP (mg/L)	11.2 ± 10.3	5.6 ± 6.3	0.016
PCT (ng/mL)	10.9 ± 23.1	2.8 ± 7.3	0.094
WBC (× 10^9^/L)	8.9 ± 3.7	11.4 ± 11.0	0.191
Serum creatinine (mg/dL)	2.9 ± 2.0	2.2 ± 1.4	0.136

Continuous variables are expressed as mean ± standard deviation. C-reactive protein (CRP) levels were significantly higher in the AGPN group, while no statistically significant differences were observed for procalcitonin (PCT), white blood cell count (WBC), or serum creatinine

Microbiological findings are reported in Table 4. Gram-negative organisms predominated in both groups. Klebsiella pneumoniae was the most frequently isolated pathogen among patients with AGPN (52.4% vs. 28.9% in patients without AGPN), although the difference did not reach statistical significance (p = 0.099). Escherichia coli was isolated with similar frequency in the two groups (23.8% vs. 22.2%, p = 1.000). Other microorganisms were less frequently isolated, and no statistically significant differences in the distribution of individual pathogens were observed between groups.

**Table 4:** Distribution of microbiological isolates in patients with and without acute graft pyelonephritis (AGPN)

Pathogen	AGPN (N=21)	No AGPN (N=45)	P-Value
*Escherichia coli*	5 (23.8%)	10 (22.2%)	1.000
*Klebsiella pneumoniae*	11 (52.4%)	13 (28.9%)	0.099
*Proteus Mirabilis*	2 (9.5%)	1 (2.2%)	0.236
*Enterococcus Faecalis*	2 (9.5%)	2 (4.4%)	0.587
*Enterococcus Faecium*	2 (9.5%)	0 (0.0%)	0.098
*Pseudomonas Aeruginosa*	2 (9.5%)	3 (6.7%)	0.650
*Staphylococcus Aureus*	0 (0.0%)	1 (2.2%)	1.000
*Candida* SPP.	0 (0.0%)	1 (2.2%)	1.000
*Staphylococcus Epidermidis*	1 (4.8%)	1 (2.2%)	0.538

Data are presented as numbers (percentages). Gram-negative bacteria were the predominant pathogens in both groups, particularly Klebsiella pneumoniae and Escherichia coli, with no statistically significant differences between groups

### US findings

On conventional B-mode ultrasound (Table 5), several findings were significantly more frequent in patients with AGPN than in those without AGPN. These included globular appearance (14.3% vs. 0.0%, p = 0.029), cortical hyperechogenicity (23.8% vs. 0.0%, p = 0.002), cortical hypoechogenicity (28.6% vs. 4.4%, p = 0.010), perirenal fluid collection (38.1% vs. 11.1%, p = 0.018), and ureteral wall thickening (42.9% vs. 8.9%, p = 0.002). Conversely, increased or reduced kidney size, hydroureteronephrosis, bladder sediment, bladder distension, and renal calculi did not differ significantly between groups. CEUS demonstrated high diagnostic performance for the detection of AGPN. CEUS was positive in 19 of the 21 patients with a confirmed diagnosis of AGPN, with two false-negative results and no false-positive findings among the 45 patients without AGPN. This corresponded to a sensitivity of 90.5% (95% CI 69.6–98.8%), specificity of 100% (95% CI 92.1–100%), positive predictive value (PPV) of 100% (95% CI, 82.4–100%), and negative predictive value (NPV) of 95.7% (95% CI 85.5–99.5%). The positive likelihood ratio (LR +) tended to infinity because no false-positive results were observed, whereas the negative likelihood ratio (LR −) was 0.10. Considering CEUS as a binary diagnostic test, the area under the receiver operating characteristic curve was 0.95. Most CEUS-detected lesions were unifocal, whereas multifocal involvement was observed in a minority of cases.

**Table 5:** Conventional ultrasound findings in patients with and without acute graft pyelonephritis (AGPN)

Ultrasound finding	AGPN (N=21)	No AGPN (N=45)	P-value
Increased kidney size	4 (19.0%)	6 (13.3%)	0.714
Reduced kidney size	2 (9.5%)	1 (2.2%)	0.236
Globular appearance	3 (14.3%)	0 (0.0%)	0.029
Cortical hyperechogenicity	5 (23.8%)	0 (0.0%)	0.002
Cortical hypoechogenicity	6 (28.6%)	2 (4.4%)	0.010
Perirenal fluid collection	8 (38.1%)	5 (11.1%)	0.018
Hydroureteronephrosis	2 (9.5%)	4 (8.9%)	1.000
Ureteral wall thickening	9 (42.9%)	4 (8.9%)	0.002
Bladder Sediment	2 (9.5%)	0 (0.0%)	0.098
Bladder distension	2 (9.5%)	0 (0.0%)	0.098
Renal calculi	2 (9.5%)	2 (4.4%)	0.587

Data are presented as number (percentage). Percentages were calculated using the total number of patients in each group as denominator. Comparisons between groups were performed using Fisher’s exact test

**Fig. 2:**
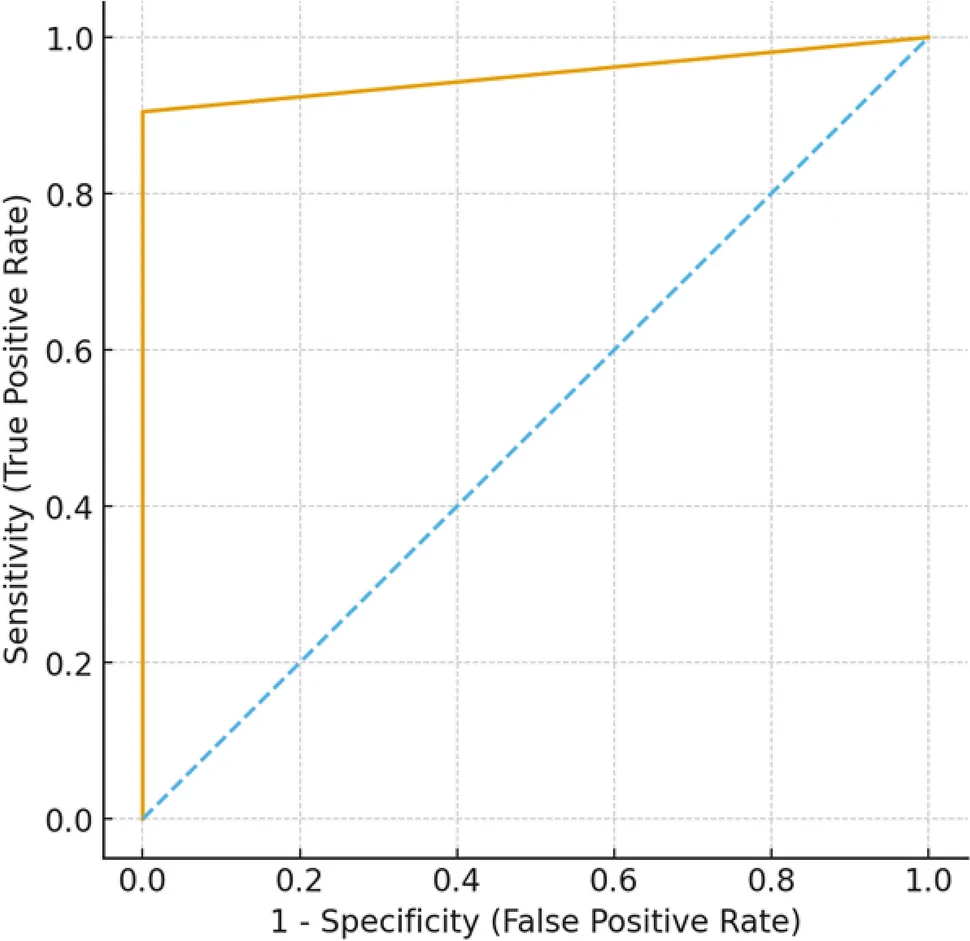
Receiver operating characteristic (ROC) curve of contrast-enhanced ultrasound (CEUS) for the diagnosis of acute graft pyelonephritis in renal transplant recipients. CEUS demonstrated excellent diagnostic accuracy, with an area under the curve (AUC) of 0.95, sensitivity of 90.5%, and no false-positive results (specificity 100%)

CECT was available in a minority of the study population; in particular, six patients (9.1%) underwent CECT. Given the non-systematic use of CECT and the heterogeneous temporal relationship between CECT and CEUS, a formal head-to-head diagnostic accuracy analysis was not considered appropriate. The two false-negative CEUS cases subsequently underwent CECT, which demonstrated findings consistent with acute pyelonephritis. The first patient was a 37-year-old woman presenting with fever and flank pain, with elevated inflammatory markers (CRP 34.41 mg/L, PCT 0.9 ng/mL, and WBC 16.47 ×10⁹/L); CEUS was performed 5 days after symptom onset. The second patient was a 73-year-old woman presenting with flank pain, with CRP 19.85 mg/L, PCT 0.2 ng/mL, and WBC 11.9 ×10⁹/L; CEUS was performed 1 day after symptom onset. Escherichia coli was isolated in both cases. Conventional ultrasound showed no major parenchymal abnormalities in the first patient, whereas increased graft size and hydroureteronephrosis were observed in the second. In both patients, CEUS was negative, while subsequently performed CECT demonstrated findings consistent with acute pyelonephritis. Given the limited number of cases and the non-standardized timing between CEUS and CECT, these observations were considered descriptive and no formal comparative analysis was performed (Fig. 2).

## Discussion

In this retrospective study, CEUS demonstrated high diagnostic accuracy for the detection of AGPN in renal transplant recipients, with a sensitivity of 90.5% and a specificity of 100%. The absence of false-positive findings resulted in a PPV of 100%, while the NPV was 95.7%. These findings suggest that CEUS may represent a valuable imaging modality for assessing suspected AGPN, particularly when accurate characterization of graft parenchymal involvement is required. Renal transplant recipients represent a high-risk population for infectious complications because of chronic immunosuppression, urological manipulation, and post-transplant anatomical alterations. In this setting, the diagnosis of AGPN may be challenging because clinical manifestations can be nonspecific or attenuated. In our cohort, however, fever was significantly more frequent among patients with confirmed AGPN, whereas flank pain and urinary symptoms, although numerically more frequent, did not significantly differ between groups. This finding highlights the importance of integrating clinical presentation with laboratory, microbiological, and imaging findings rather than relying on individual symptoms alone.

Conventional ultrasound remains an important first-line examination in renal transplant recipients because of its widespread availability, repeatability, and ability to assess graft morphology, urinary tract abnormalities, and vascular complications. Nevertheless, conventional B-mode findings of parenchymal infection are generally considered nonspecific. In our cohort, several ultrasound abnormalities were significantly more frequent in patients with AGPN, including globular appearance, cortical hyperechogenicity, cortical hypoechogenicity, perirenal fluid collection, and ureteral wall thickening. Among these, cortical echogenicity abnormalities and ureteral wall thickening showed particularly strong associations with AGPN. Although none of these findings can be considered diagnostic in isolation, their presence may increase the suspicion of graft parenchymal involvement and support further evaluation with CEUS.

CEUS provides additional information by enabling real-time assessment of renal microvascular perfusion [10]. In acute pyelonephritis, inflammatory parenchymal involvement may result in focal or multifocal areas of reduced perfusion, which can be visualized as hypoenhancing or non-enhancing regions relative to the surrounding renal parenchyma. In our study, CEUS correctly identified 19 of 21 patients with AGPN, with only two false-negative examinations and no false-positive findings. These results are consistent with previous studies reporting a high diagnostic performance of CEUS for acute pyelonephritis and its ability to identify parenchymal abnormalities that may be poorly visualized on conventional ultrasound [11–15]. An additional advantage of CEUS in renal transplant recipients is its favorable safety profile. Ultrasound contrast agents are not nephrotoxic and CEUS does not involve ionizing radiation, making it particularly attractive in patients with impaired graft function and in those requiring repeated imaging examinations. These characteristics may be especially relevant in renal transplant recipients, in whom preservation of renal function and limitation of unnecessary exposure to iodinated contrast media are important considerations.

From a microbiological perspective, Gram-negative organisms predominated in our cohort. Klebsiella pneumoniae was the most frequently isolated pathogen among patients with AGPN, followed by Escherichia coli. Although K. pneumoniae was numerically more frequent in patients with AGPN than in those without AGPN, the difference did not reach statistical significance. Overall, no significant differences in the distribution of individual microorganisms were observed between groups.

Taken together, our findings suggest that CEUS may represent a valuable imaging option in the diagnostic work-up of renal transplant recipients with suspected AGPN, complementing conventional ultrasound and potentially reducing the need for contrast-enhanced CT in selected patients. However, the very high diagnostic performance observed in our cohort should be interpreted cautiously. The retrospective, single-center design and relatively small sample size limit the generalizability of the findings. Moreover, a uniform independent imaging reference standard was not available, as contrast-enhanced CT was performed only when clinically indicated and the final diagnosis was based on the integration of clinical, laboratory, microbiological, and available imaging findings. This approach may have introduced classification and incorporation bias, particularly if CEUS findings contributed to the final clinical diagnosis. In addition, all CEUS examinations were performed by a single experienced operator, which may have optimized diagnostic performance and limits assessment of interobserver reproducibility. Finally, systematic follow-up imaging was not available in all patients. Prospective multicenter studies using standardized diagnostic criteria, independent reference standards, and blinded CEUS interpretation are warranted to validate these findings, assess interobserver reproducibility, and better define the position of CEUS within diagnostic algorithms for suspected AGPN in renal transplant recipients.

## Study limitations

This study has several limitations that should be acknowledged. First, its retrospective design may have introduced selection bias, as CEUS was performed only in patients with clinical suspicion of acute pyelonephritis. Second, the sample size was relatively small, potentially limiting the statistical power of the analysis. Moreover, a uniform independent imaging reference standard was not available, as CECT was performed only when clinically indicated and the final diagnosis was based on the integration of clinical, laboratory, microbiological, and available imaging findings. This reflects real-world clinical practice in renal transplant recipients, in whom contrast-enhanced CT may be deliberately avoided, particularly in patients with severe graft dysfunction, to limit exposure to iodinated contrast media. Consequently, systematically requiring CECT as the reference standard would have been neither clinically appropriate nor feasible in a substantial proportion of this population. Importantly, however, clinicians establishing the final diagnosis were not systematically blinded to CEUS findings. Therefore, CEUS may have contributed to the final diagnostic classification in some patients, introducing potential incorporation bias. Additionally, all CEUS examinations were performed and interpreted by a single experienced operator, and complete standardized digital recordings suitable for independent blinded reassessment were not available for all patients. Consequently, inter-observer agreement could not be assessed. This operator dependency represents an important limitation and may restrict the reproducibility and generalizability of the observed diagnostic performance. Finally, the lack of systematic follow-up imaging may have led to underestimation of subclinical or resolving lesions [16, 17].

## Conclusions

CEUS showed high diagnostic accuracy for the detection of AGPN in renal transplant recipients, with excellent specificity and a high negative predictive value. Its favorable safety profile, absence of nephrotoxicity and ionizing radiation, and ability to provide real-time assessment of graft parenchymal perfusion make CEUS a valuable imaging option in patients with suspected acute graft pyelonephritis. CEUS may complement conventional ultrasound and potentially reduce the need for contrast-enhanced CT in selected patients, particularly when renal function is impaired or repeated imaging is required. However, given the retrospective single-center design and the limited sample size, these findings should be considered preliminary. Larger prospective multicenter studies using standardized and independent reference standards are warranted to validate the diagnostic performance of CEUS and better define its role within diagnostic algorithms for acute graft pyelonephritis.

## Data Availability

Data are available upon request from the authors.
